# Case Report: Functional validation of a *PKD1* c.7489 + 5G>A variant in an ADPKD family

**DOI:** 10.3389/fgene.2026.1850997

**Published:** 2026-08-06

**Authors:** Qiong Pan, Yuefang Liu, Xueping Sun, Shoulian Lu, Lingling Li, Jiandong Shen

**Affiliations:** 1 Genetic and Prenatal Diagnosis Center, Huai’an Maternal and Child Health Care Hospital Affiliated to Yangzhou University, Huai’an, Jiangsu, China; 2 Genetic and Prenatal Diagnosis Center, The Huai’an Maternity and Child Clinical College of Xuzhou Medical University, Huai’an, Jiangsu, China; 3 Department of Medical Technology, Jiangsu College of Nursing, Huai’an, Jiangsu, China; 4 State Key Laboratory of Reproductive Medicine and Offspring Health, Clinical Center of Reproductive Medicine, The First Affiliated Hospital with Nanjing Medical University, Nanjing, Jiangsu, China

**Keywords:** autosomal dominant polycystic kidney disease (ADPKD), minigene assay, *PKD1* gene, preimplantation genetic testing for monogenic disorders (PGT-M), splicing variant

## Abstract

**Background:**

Autosomal dominant polycystic kidney disease (ADPKD) is most commonly caused by pathogenic variants in *PKD1*. Here, we reported the functional characterization of an intronic *PKD1* variant identified in an ADPKD-affected family and its subsequent application in preimplantation genetic testing for monogenic disorders (PGT-M).

**Case presentation:**

A three-generation ADPKD family was enrolled. Whole-exome sequencing revealed a heterozygous *PKD1* c.7489 + 5G>A variant, which co-segregated with the disease and was initially classified as a variant of uncertain significance. A minigene assay demonstrated that the variant induced skipping of exon 18, leading to a frameshift (p.Arg2404Valfs*123), supporting its reclassification as pathogenic. The couple underwent PGT-M using trophectoderm biopsy, haplotype linkage analysis, and direct mutation detection. An unaffected pregnancy was achieved, and subsequent prenatal diagnosis confirmed the absence of the variant and a normal chromosomal karyotype.

**Conclusion:**

We validated a pathogenic splicing variant in *PKD1* and successfully applied PGT-M to prevent disease transmission, resulting in an unaffected pregnancy.

## Introduction

Primary cilia are the antenna-like organelles formed by a microtubule-based skeleton on the surface of almost all polar cells including renal tubular cells (Mill et al., 2023). Primary cilia plays important roles in tissue morphogenesis, and chemical and mechanical signal transduction. Alterations in their function cause a broad range of developmental diseases collectively called the ciliopathies characterized by various phenotype affecting one or multiple organs (McConnachie et al., 2021). If kidney involvement is the predominant clinical feature, we define the disorder as a renal ciliopathy (McConnachie et al., 2021; Devlin et al., 2025). Renal ciliopathies are characterized by both common cyst formation and strikingly diverse phenotype, with polycystic kidney disease (PKD) and nephronophthisis (NPH) being the two most prevalent forms (Boletta and Caplan, 2025; Devlin et al., 2025). Autosomal dominant PKD (ADPKD) is characterized by the formation of bilateral renal cysts, resulting in a progressive increase in kidney volume, which affects up to 0.1%–0.25% individuals worldwide and leads to end stage renal disease (ESRD) in ∼70% of affected individuals (Orisio et al., 2024). Other renal manifestations can include flank pain, haematuria, stones, and polyuria.

ADPKD results from disease-causing variants in either the gene *PKD1* (polycystin 1, PC1) or *PKD2* (polycystin 2, PC2), accounting for about 78% and 13%, respectively (Hopp et al., 2020). Polycystin-1 is a protein of 4303 amino acids encoded by the 46 exons of the *PKD1* gene (Boletta et al., 2001). The 3′-terminal region spanning exons 32 to 46 exists as a single-copy segment whereas the 5′-terminal region comprising exons 1 to 32 is repeated six times on chromosome 16, giving rise to six pseudogenes. These pseudogenes share approximately 97.7% sequence identity with the functional gene (Symmons et al., 2008). *PKD1* presents a high rate of mutations without mutation hotspots, partly due to the large size of the gene and due to possible gene conversion events between the functional *PKD1* gene and pseudogenes. Therefore, the molecular diagnosis of ADPKD is extremely difficult because of the complexity of the *PKD1* gene.

At present, there is no effective treatment for ADPKD patients. Hence, preventing the transmission of mutated *PKD1* gene to their offspring is of great importance. For individuals with a confirmed pathogenic *PKD1* variant and a comprehensive family medical history, preimplantation genetic testing for monogenic disorders (PGT-M) combined with single nucleotide polymorphism (SNP) linkage analysis among pedigree members is one of the established strategies to block disease transmission. In this report, we first identified the *PKD1* c.7489 + 5G>A variant in intron 18 as the underlying genetic cause of ADPKD in this family. Subsequently, the couple underwent PGT-M, and a noncarrier embryo was transferred, achieving an ongoing pregnancy. Finally, prenatal diagnosis confirmed the absence of the familial *PKD1* variant and normal chromosomal status.

## Materials and methods

### Patient

A Chinese family with a history of ADPKD was recruited for this study at the Reproductive Medicine Center of the First Affiliated Hospital of Nanjing Medical University in August 2024. The family included three affected individuals and eight healthy members. The proband, a 32-year-old female with no prior history of childbirth, presented together with her husband for fertility counseling due to her positive family history of PKD.

Whole-exome sequencing (WES) was performed on genomic DNA extracted from the proband’s blood sample. For segregation analysis, additional blood samples were obtained from the proband’s affected father and younger brother, as well as from five healthy family members (mother, grandmother, uncle, elder uncle, and aunt). Genomic DNA was extracted using a blood genomic column-based small-scale extraction kit (Chaozhou Kaipu, China). All participants provided written informed consent. The research protocol was approved by the local ethics committee of The First Affiliated Hospital of Nanjing Medical University (2018-SR-063. A1).

### Genetic analysis

Sample libraries of gDNA were prepared using the TruSeq DNA Sample Preparation kit (Illumina Inc., San Diego, CA, United States) and the exome regions were enriched using the FindRare focused exome probes v1 kit (FindRare Inc., Beijing, China) according to manufacturer’s protocols. Paired-end short-read sequencing was performed on an Illumina platform with a PE150 read length (Illumina Inc., San Diego, CA, United States).

Bioinformatics analyses were performed using an in-house pipeline. Briefly, sequencing reads that passed quality filtering were aligned with the human reference genome, hg19, using the Burrows-Wheeler Aligner program. Variants were identified using a combination of the FreeBayes, Genome Analysis Toolkit, CNVnator and in-house software programs. All the mutations were compared with databases such as dbSNP, ExAC or gnomAD and the frequency of occurrence were then calculated in different population group. Predicted pathogenicity data were obtained using the SNPEff, Ensembl Variant Effect Predictor, Sorting Intolerant From Tolerant, Polymorphism Phenotyping version 2 and dbscSNV databases. *PKD1* gene and *PKD2* gene were mainly focused, and the average sequencing depth was >100X. The deleteriousness of this variant was assessed according to American College of Medical Genetics (ACMG) 2015 standards and guidelines.

### Long range-polymerase chain reaction and sanger validation

It has been reported that long-range (LR)-PCR overcomes interference from highly homologous *PKD1* pseudogenes via two core strategies (Tan et al., 2012). First, all nine pairs of LR-PCR primers are computationally designed to anchor at unique genomic sites with rare mismatches between authentic *PKD1* and its homologs. Second, the 2–6 kb amplicon size acts as a natural size filter. Pseudogenes cannot generate target-length products even with partial non-specific primer annealing. Since our variant was located in intron 18, the fragment including exon 15–21 was amplified in a distinct LR-PCR using PCR primers. The LR-PCR primer sequences were listed in Table 1. LR-PCR was performed using the PCR amplification system listed in Table 2. The PCR amplification conditions are as follows: 95 °C for 3 min, followed by 35 cycles of 95 °C for 30 s and 68 °C for 4.5 min, with a final extension step at 72 °C for 10 min. To cover the c.7489 + 5G>A variant in intron 18, the LR-PCR products of *PKD1* were then purified and sequenced with specific forward primer located at 98 bp downstream of exon 17 and reverse primer located at 55 bp upstream of intron 18. Primer sequences were listed in Table 1. All the primers, PCR amplification system and amplification condition used in this study were obtained from the literature (Tan et al., 2012).

**TABLE 1:** LR-PCR and sanger sequencing primers.

Primers name	Sequence (5′→3′)
PKD1-E15/21F	ATC​CCT​GGG​GGT​CCT​ACC​ATC​TCT​TA
PKD1-E15/21R	ACA​CAG​GAC​AGA​ACG​GCT​GAG​GCT​A
PKD1-E17/18F	GAA​ACC​TGG​AGT​TTG​GGA​GCA​GC
PKD1-E17/18R	TGACGTCACAGAGTCGGG

**TABLE 2:** LR-PCR amplification system.

Agent	Volume
5 × STAR buffer I	10 μL
2.5 mM dNTP	8 μL
PrimeSTAR GXL	1 μL
5M betaine	5 μL
DMSO (10%)	2.5 μL
Sterile deionized H2O	17.5 μL
10 μM primer F	2 μL
10 μM primer R	2 μL
50ng/ul g DNA	2 μL

### Blood RNA extraction and reverse transcription PCR

Blood RNA was extracted and enriched. Reverse transcription PCR (RT-PCR) was performed, and the resulting cDNA was amplified using the primer pair PKD1_RT-PCR-F and PKD1_RT-PCR-R listed in Table 3. The products with 829 bp length were examined by 1% agarose gel electrophoresis and further confirmed by Sanger sequencing.

**TABLE 3:** Primers for blood cDNA amplification.

Primers name	Sequence (5′→3′)
PKD1_RT-PCR-F	CAG​GCC​GTG​TAC​GAA​GTG​AG
PKD1_RT-PCR-R	ACA​CCA​GAG​TCT​CCG​TGA​TG

### Minigene splicing assay

Two pairs of nested primers (28114-PKD1-F and 30200-PKD1-R, 28469-PKD1-F and 29898-PKD1-R) were designed for nested PCR by using genomic DNA of the proband as template. Two minigenes namely pcDNA3.1-PKD1-wild type (wt) and pcDNA3.1-PKD1-mutant (mut) were constructed as below. All primer sequences were shown in Table 4.

**TABLE 4:** Vector construction and minigene primers.

Primers name	Sequence (5′→3′)
28114-PKD1-F	TGT​TGC​TGG​ATG​TGG​TGG​GC
28469-PKD1-F	CCT​CAG​TGA​GCA​TTG​CCT​GC
29898-PKD1-R	CAG​GCA​GGG​GTA​CAG​GTC​TT
30200-PKD1-R	GGC​ACA​GCA​AGC​TGT​CAG​CA
pcDNA3.1-PKD1-Kpnl-F	GCT​TGG​TAC​CAT​GGT​GCT​GAT​CCG​GAG​TGG​CCG
pcDNA3.1-PKD1-Ecol-R	TGC​AGA​ATT​CCC​TGT​TGA​GGG​CGA​CCA​CAG​C
pcDNA3.1-F	CTA​GAG​AAC​CCA​CTG​CTT​AC
pcDNA3.1-R(BGH-R)	TAGAAGGCACAGTCGAGG

### The strategy of pcDNA3.1-PKD1-wt/mutant plasmids

By using nested PCR product as template, the target gene region covering Exon17 (144bp)- Intron17 (127bp)-Exon18 (280bp)- Intron18 (93bp)-Exon19 (214bp) of PKD1 was amplified by primers pcDNA3.1-PKD1-KpnI-F and pcDNA3.1-PKD1-Ecol-R. The obtained products were inserted into the pcDNA3.1 vector and monoclonal expression of pcDNA3.1-PKD1-wt and pcDNA3.1-PKD1-mutant were selected. Subsequently, the plasmids were validated through Sanger sequencing.

### Transfection

HEK-293T cells were maintained as monolayer cultures in Dulbecco’s modified Eagle’s medium (Life Co., United States) supplemented with 10% fetal bovine serum (Gibco, 10099–141), 20 U/mL penicillin and 20 μg/mL streptomycin (Sigma-Aldrich), with a 5% CO2 concentration at 37 °C. For transient transfection, cells were seeded into six wells. After reaching >70% confluence at 12–24 h, culture medium was abolished and fresh culture medium without penicillin and streptomycin was added. Then, cells were transfected with pcDNA3.1-PKD1-wt or pcDNA3.1-PKD1-mutant plasmids using Lipofectamine 2000 Reagent (Invitrogen, Carlsbad, United States) following the manufacturer’s instructions.

### Cell RNA extraction and reverse transcription PCR

With total RNA extracted and reverse-transcribed into cDNA after 48 h of transfection, reverse transcription PCR (RT-PCR) was performed. Finally, the cDNA was amplified by a primer pair of pcDNA3.1-F and pcDNA3.1-R (BGH-R). The products were examined using 1% agarose gel electrophoresis and further confirmed by Sanger sequencing. To substantiate the conclusion drawn in this study, the assay was repeated in HeLa cells by using the same experimental method as above.

### PGT-M and prenatal diagnosis

Ovarian stimulation, *in vitro* fertilization, trophectoderm (TE) biopsy, and later embryo transfer processes after genetic testing were conducted in our reproductive medical center as previously described (Cai et al., 2012; Wang et al., 2013). Genomic DNA from biopsied cells was amplified using a multiple displacement amplification kit REPLI-g (Qiagen, Germany). Firstly, copy number variations (CNV) analysis was performed via next-generation sequencing (NGS). Library preparation was carried out with the VeriSeq-MiSeq Kit (Illumina, United States), followed by low-depth whole-genome sequencing on the MiSeq platform (MiSeqDx Universal Kit V3 SBS, United States). Raw sequencing data was analyzed on a cloud-based platform (PGXCloud, available at www.pgxcloud.com), applying a bioinformatics algorithm consistent with a previous report (Fiorentino et al., 2014). With this method, deletions and duplications not smaller than 4 Mb could be detected (the detection limit). Subsequently, Sanger sequencing validation of biopsied cells for *PKD1* c.7489 + 5G>A variant was conducted by the method “long range-polymerase chain reaction and Sanger validation”. To avoid allele dropout, haplotype analysis is essential to avoid the misdiagnoses resulting from possible allele dropout in direct detection. The Infinium Asian Screening Array-24 Kit (Illumina, United States) SNP array was used for haplotyping based on the principle described in the previous literature (Handyside et al., 2010). A total of 103 SNPs selected for the haplotype analysis were listed in Supplementary Table S1. Of the 103 SNP markers analyzed, no SNPs were located within the *PKD1* gene, 47 SNPs flanked the 5′side, and 56 SNPs flanked the 3′side of the gene.

To further verify the absence of *PKD1* c.7489 + 5G>A variant and chromosomal normality, the prenatal diagnosis was conducted at the 18th week of gestation by amniocentesis. About 40 mL of amniotic fluid was removed by aspiration and extracted for fetal genomic DNA using Thermo Fisher MagMAX™ DNA Multi-Sample Ultra 2.0 Kit (A36570, Thermo Fisher). Sanger sequencing validation of fetal DNA for *PKD1* c.7489 + 5G>A variant was conducted as method “long range-polymerase chain reaction and Sanger validation”. Chromosome testing was hybridized with chromosomal microarray analysis (CMA) (Affymetrix CytoScan 750K array) by following the manufacturer’s protocol. The chip data in CEL file were analyzed by Chromosome Analysis Suite (CHAS) software (v2.0) to produce CYCHP file. The CNVs in CYCHP file were called by CHAS.

## Results

### Clinical phenotype of affected members in ADPKD family

**FIGURE 1:**
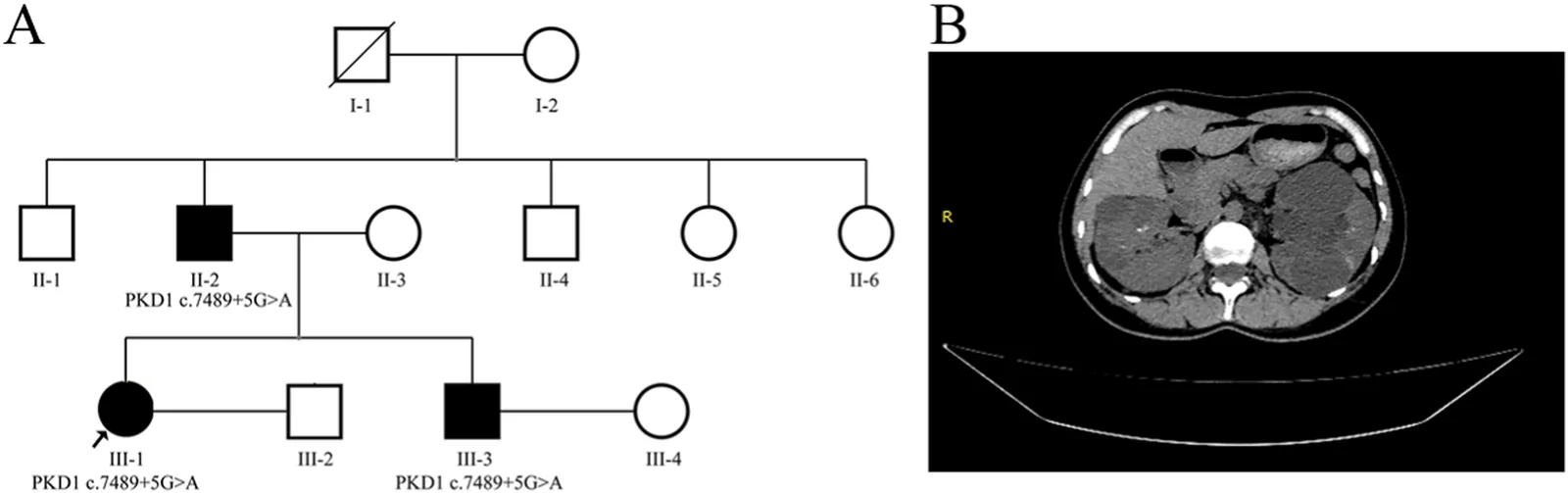
The pedigree and phenotype of the ADPKD family **(A)** The pedigree of the ADPKD family spanned three generations with three affected members; **(B)** Bilateral multiple renal cysts (maximum diameter 5.5 cm), multiple stones, and hydronephrosis of the proband detected by computed tomography (CT).

A family with ADPKD, including ten individuals from three generations, was studied. According to the pedigree (Figure 1A), three patients in the family have been found to have multiple renal cysts on ultrasound, including proband (III-1), the proband’s father (II-2) and the proband’s younger brother (III-3). Polycystic kidney disease in the proband was incidentally detected via ultrasound during a routine physical examination at age 37. Subsequent computed tomography (CT) confirmed bilateral multiple renal cysts (maximum diameter 5.5 cm), multiple renal calculi, and left hydronephrosis (Figure 1B), with preserved renal size (right kidney: 175 mm*84 mm*66 mm; left kidney: 145 mm*100 mm*78 mm), morphology, and clinical renal function. Urinalysis indicated positive occult blood test. Renal function tests showed normal serum creatinine and estimated glomerular filtration rate (eGFR) (Table 5). The main extrarenal manifestation was multiple liver cysts (Table 5). Height-adjusted total kidney volume (HtTKV), a renal prognosis prediction model for ADPKD, was 671m1/m.

**TABLE 5:** Clinical phenotypes of affected family members.

Patient	Sex	Age (year)	Height (mm)	Renal function	Kidney imaging	HtTKV	Urinalysis	Extra-renal phenotypes
II-2	Male	60	170	ESRD (diagnosed at 50 years)	Ultrasound imaging at 60 years revealed markedly enlarged bilateral kidneys (right kidney:199 mm*90 mm left kidney:162 mm*94 mm), accompanied by multiple renal cysts (maximum diameter 4.3 cm) and renal calculi	937 mL/m	No urine production	—
III-1	Female	37	164	eGFR: 110 mL/min; serum creatinine:61.2 μmol/L (References 41.0–73.0)	Computed tomography (CT) at 37 years confirmed bilateral multiple renal cysts (maximum diameter 5.5 cm), multiple renal calculi, and left hydronephrosis, with preserved renal size (right kidney 175 mm*84 mm*66 mm left kidney: 145 mm*100 mm*78 mm), morphology, and clinical renal function	671 mL/m	Occult blood test:1+	Multiple liver cysts
III-3	Male	30	172	Unknown	Ultrasound examination at age 32 showed normal renal size and morphology (right kidney 155 mm*55 mm*75 mm left kidney: 140 mm*64 mm*70 mm), with bilateral multiple renal cysts (maximum diameter 2.5 cm) and renal calculi.	386 mL/m	—	—

eGFR, estimated glomerular filtration rate; ESRD, end-stage renal disease.

The proband’s father (II-2) was diagnosed with end-stage renal disease (ESRD) at age 50 and commenced renal dialysis. Polycystic kidney disease was also detected at that time. At age 60, ultrasound imaging of the proband’s father (II-2) revealed markedly enlarged bilateral kidneys (right kidney:199 mm*90 mm; left kidney:162 mm*94 mm), accompanied by multiple renal cysts (maximum diameter 4.3 cm) and renal calculi (Table 5). HtTKV was 937m1/m. No extrarenal manifestations were clearly recorded.

Ultrasound examination of the proband’s younger brother (III-3) at age 32 showed bilateral multiple renal cysts (maximum diameter 2.5 cm) and renal calculi despite normal renal morphology and size (right kidney: 155 mm*55 mm*75 mm; left kidney: 140 mm*64 mm*70 mm) (Table 5). No hydronephrosis or perinephric effusion was observed. Urinalysis, renal function and extrarenal phenotypes were not recorded. HtTKV was 386m1/m.

### Identification of an intron variant of *PKD1* gene in the family

**FIGURE 2:**
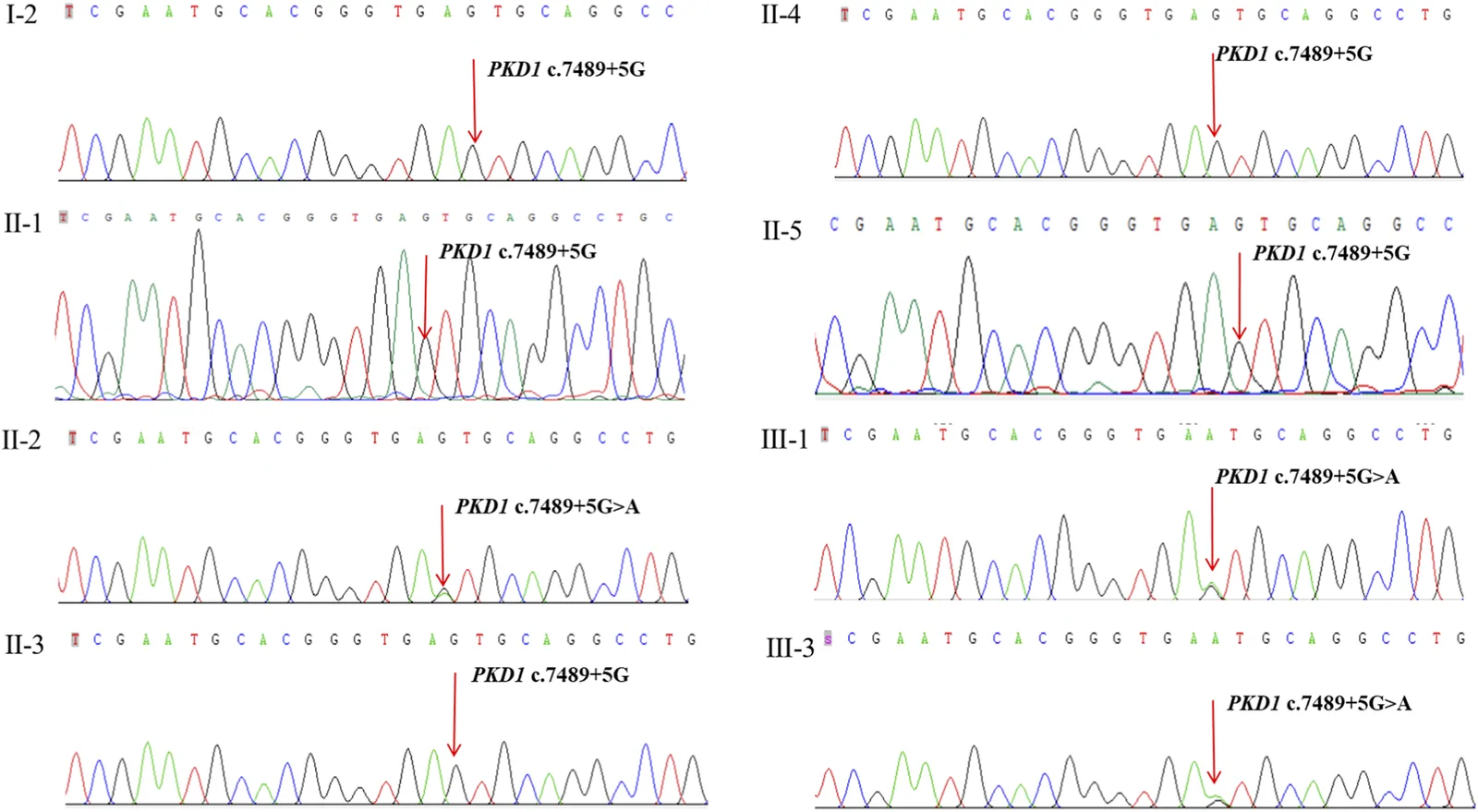
Family segregation analysis of *PKD1* c.7489 + 5G>A variant. *PKD1* c.7489 + 5G>A variant was detected in proband (III-1), the proband’s father (II-2) and the proband’s younger brother (III-3) but not detected in mother (II-3), grandmother (I-2), uncle (II-4), elder uncle (II-1), and aunt (II-5) by Sanger sequencing.

Exome sequencing was performed for mutation screening in the proband. Bioinformatics analysis of the VCF file identified a total of 17,566 variants. After filtering out clearly benign variants, 3,758 variants remained. Based on the patient’s characteristic clinical phenotype, we prioritized the *PKD1* and *PKD2* genes, which led to the retention of only five variants in *PKD1* (NM_001009944.2): c.7489 + 5G>A, c.1849 + 10T>G, c.1849 + 14T>G, c.1849 + 17T>G, and c.1849 + 21T>G. Among these, c.7489 + 5G>A was predicted by *in silico* tools to be the variant most likely to affect splicing. This variant has previously been described in three PKD patients (Mallawaarachchi et al., 2021; Hort et al., 2023) (PS4-Moderate) and was absent both in the 1000 Genomes Project and the ExAC databases (PM2_Supporting). Since *PKD1* has high homology with pseudogenes, LR-PCR and Sanger sequencing were performed to validate the variant. The results showed that the proband, the proband’s father and proband’s brother all carried this variant (Figure 2). Other five healthy members including mother (II-3), grandmother (I-2), uncle (II-4), elder uncle (II-1), and aunt (II-5) did not have the variant (Figure 2) (PP1). This variant was classified as a variant of uncertain significance (VUS) according to the ACMG criteria (PM2_Supporting + PP1 + PP4 + PS4-Moderate).

### The c.7489 + 5G>A variant in *PKD1* induced skipping of exon 18

The variant c.7489 + 5G>A of *PKD1* is located at the +5 position of the donor splice site of intron 18. Notably, a study by Hort et al. (Hort et al., 2023) demonstrated that *PKD1* c.7489 + 5G>A caused partial retention of intron 18. Accordingly, we replicated the experimental strategy adopted in the previous study by performing Sanger sequencing on cDNA reverse-transcribed from peripheral blood total RNA of the patient. Nevertheless, no aberrant splicing event was identified in the peripheral blood-derived cDNA (Supplementary Figure S1). Given the potential limitations and confounding factors of blood RNA detection, we further conducted an *in vitro* minigene splicing assay for functional verification.

**FIGURE 3:**
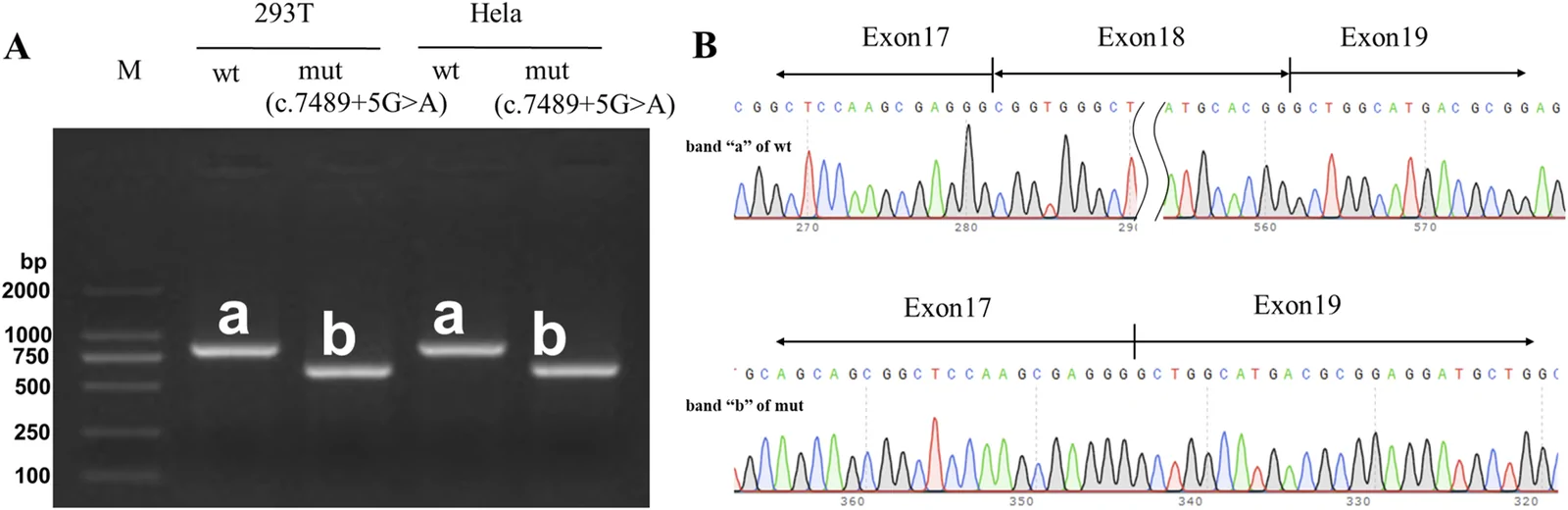
The *in vitro* minigene assay of wild type (wt) and *PKD1* c.7489 + 5G>A (mut) variant. **(A)** Gel electrophoresis confirmed that the only band “a” was detected in wt minigene with the size between 750 bp to 1,000 bp. The only band “b” was detected in mut minigene with the size between 500 bp to 750 bp; **(B)** Sanger sequencing confirmed that band “a” was a normal transcript including exon17, exon 18 and exon 19. However, the c.7489 + 5G>A variant led to exon 18 skipping.

Wild-type (wt) and mutant (mut, *PKD1* c.7489 + 5G>A) minigene constructs were generated as described in method “Minigene splicing assay”. The only band “a” was detected in wt minigene with the size between 750 bp and 1,000 bp (Figure 3A). The only band “b” was detected in mut minigene with the size between 500 bp and 750 bp (Figure 3A). Subsequent Sanger sequencing confirmed that the c.7489 + 5G>A variant induced exon 18 skipping (Figure 3B), which corresponded to the cDNA alteration c.7210_7489del and the predicted protein variant p. Arg2404Valfs*123. This variant would create a premature termination codon in exon 20, resulting in a truncated protein with a length of 2,525 amino acids. The *PKD1* c.7489 + 5G>A variant was reclassified as pathogenic according to the ACMG guidelines (PM2_Supporting + PP1+ PP4 + PS4-Moderate + PS3).

### Successful PGT-M and uncomplicated prenatal diagnosis outcome

The patient had an AMH level of 5.75 ng/mL and weighed 53 kg. She underwent two cycles of controlled ovarian stimulation. In the first cycle, a long protocol with a short-acting GnRH agonist was used, and Gonal-f (follitropin alfa) was started at 200 U. After 11 days of stimulation, 15 oocytes were retrieved, of which 14 were metaphase II (MII). ICSI yielded 10 two-pronuclei (2PN) zygotes. On day 3, there were 4 good-quality and 3 poor-quality cleavage embryos; however, extended culture failed to produce any blastocysts. In the second cycle, she followed a long protocol initiated in the early follicular phase, with 200 U of Puregon (follitropin beta). After 10 days of stimulation, an ovulation trigger was administered, and oocyte retrieval was performed 37.5 h later. A total of 14 oocytes were collected, including 10 MII oocytes. Following ICSI, 6 2PN zygotes were obtained, and on day 3, 4 good-quality and 2 poor-quality cleavage embryos were observed. Extended culture eventually resulted in 2 blastocysts. Although the patient’s ovarian reserve was expected to be normal, two cycles yielded a suboptimal number of blastocysts, with relatively low rates of oocyte maturation, fertilization, and blastocyst formation. Whether this was related to her ADPKD remains unclear. Fortunately, both blastocysts were deemed suitable for transfer after genetic testing.

Aneuploidy analysis was performed to detect chromosome CNVs including microdeletion or microduplication around 4 Mbp. While embryo E1 showed euploid embryo, embryo E2 was found to carry a 43.24-Mb deletion at chromosome 16q12.1q24.3 with a mosaicism level of 55% (Table 6). Although a total of 103 SNPs were initially selected for haplotype analysis, only 66 yielded informative genotypes. Among these 66 informative SNPs, 42 were located upstream (5′side) of the mutation site and 24 downstream (3′side). The complete set of these 66 informative SNPs is now provided as Supplementary Table S2. Finally, we confirmed that both embryos had inherited the unaffected haplotype (Supplementary Table S2). This result was further validated by Sanger sequencing, which demonstrated the absence of the pathogenic *PKD1* variant (c.7489 + 5G>A) in both embryos (Supplementary Figure S2). Based on these findings, embryo E1 having a superior blastocyst grade (5AB) was selected for transfer, resulting in a ongoing pregnancy.

**TABLE 6:** Detection results summary of the two biopsied blastocysts.

Number	Blastocysts score	Haplotype analysis	Sequencing (c.7489 + 5)	CNV analysis
E1	5AB	WT	WT	Euploid
E2	5BB	WT	WT	del (mosaic) (16) (q12.1q24.3) (55%)

At the 18th week of gestation, prenatal diagnosis was performed. The absence of the variant and normal chromosomal status was confirmed. The newborn was a full-term male delivered by cesarean section at 41 weeks of gestation, with a birth weight of 4400 g. Prenatal and postnatal renal ultrasound examinations showed normal renal size, renal parenchymal echo and structure, with no renal cysts detected. As of the submission date, the infant was 1 year old and maintained normal physical growth and development.

## Discussion

RNA splicing is a critical post-transcriptional process that precisely removes introns from precursor messenger RNA (pre-mRNA) to generate a contiguous coding sequence for protein translation (Bonnal et al., 2020). Alterations in pre-mRNA splicing have long been recognized as a potential mechanism underlying rare genetic diseases. Approximately half of disease-associated single-nucleotide variants are now estimated to disrupt pre-mRNA splicing, highlighting the importance of functional RNA studies in variant interpretation (Strauch et al., 2022; Liu et al., 2023). Splicing fidelity depends on the coordinated interaction between *cis*-regulatory elements and *trans*-acting factors. The *cis*-elements are DNA sequences primarily located within introns, including the donor (5′) and acceptor (3′) splice sites, the branch point, the polypyrimidine tract, as well as splicing silencers and enhancers (Abramowicz and Gos, 2019). The trans-acting factors comprise large and highly dynamic ribonucleoprotein complexes, including five small nuclear ribonucleoprotein particles (snRNPs: U1, U2, U4, U5, and U6) (Cartegni et al., 2002; Bonnal et al., 2020).

The donor splice site, typically located at the 5′end of an intron, is evolutionarily conserved and characterized by a canonical GUAAGU motif spanning positions +1 to +6 (Vaz-Drago et al., 2017; Abramowicz and Gos, 2019). The acceptor splice site, found at the 3′end of an intron, is similarly conserved and defined by the dinucleotide AG at positions −1 to −2. Among these, the GT at the donor site and the AG at the acceptor site are absolutely invariant, serving as core splicing signals, mutations in either site consistently result in complete splicing failure or severe splicing abnormalities (Vaz-Drago et al., 2017; Abramowicz and Gos, 2019). Although not as strictly conserved as the GT dinucleotide, the AAGU motif at positions +3 to +6 within the donor site displays strong sequence preferences-for example, a marked bias for G at the +5 position (Vaz-Drago et al., 2017; Abramowicz and Gos, 2019). The U1 snRNP recognizes and binds complementarily to the conserved donor sequence. Variations within the AAGU motif can impair the binding affinity or specificity of U1 snRNP, frequently leading to reduced splicing efficiency, such as exon skipping or activation of cryptic splice sites. In *PKD1* gene, some variants affecting the +5 position at the 5′ends of *PKD1* introns such as c.2097 + 5G>A in intron five and c.2853 + 5G>A in intron 11 have been reported to disrupt normal splicing (Youn et al., 2025). The nucleotide sequence spanning positions +1 to +6 in intron 18 of *PKD1* is GTGAGT. The variant of interest altered this sequence to GTGAAT, indicating a disruption of normal splicing.

The *PKD1* c.7489 + 5G>A variant was previously shown by peripheral blood cDNA sequencing to cause partial retention of intron 18 in a 2023 study (Hort et al., 2023). However, RT-PCR and Sanger sequencing of peripheral blood RNA in the proband from our study revealed no detectable abnormal splicing of *PKD1*. In contrast to direct sequencing peripheral blood RNA, minigene assay demonstrated that this variant induced exon 18 skipping. The inconsistent splicing outcomes could be explained by several reasons. First, tissue-specific splicing regulation of *PKD1* is well recognized. Peripheral blood cells may lack essential splicing factors present in renal tissue, leading to suppressed or undetectable aberrant transcripts in blood, while the variant exerts distinct splicing defects in kidney cells. Second, low-abundance abnormal transcripts in peripheral blood may escape detection by conventional RT-PCR and Sanger sequencing, whereas the minigene system directly reflects the intrinsic splicing abnormality of the variant independent of *in vivo* regulatory networks. Third, inter-individual genetic background, haplotype differences, and variable expression of splicing modifiers may switch the splicing pattern between intron 18 retention and exon 18 skipping among different carriers. In conclusion, peripheral blood cDNA analysis alone is insufficient to fully evaluate the splicing effect of *PKD1* splice-site variants. Combined validation using peripheral blood RNA testing and *in vitro* minigene assay is recommended to comprehensively characterize splicing alterations and support pathogenicity interpretation in ADPKD.

Our minigene assay confirmed that the *PKD1* c.7489 + 5G>A splice-altering variant induced exon 18 skipping, generating an out-of-frame transcript c.7210_7489del predicted to yield p. Arg2404Valfs*123 with a premature termination codon. This molecular defect may drive ADPKD via two well-established pathogenic frameworks for *PKD1*-related disease: haploinsufficiency and the somatic two-hit cystogenesis model (Boletta and Caplan, 2025). First, the frameshift transcript may undergo nonsense-mediated mRNA decay (NMD) within renal epithelial cells, eliminating the mutant allele’s translation product and reducing total functional PC1 dosage by half—a state termed haploinsufficiency, which alone confers disease susceptibility in heterozygous carriers. Alternatively, evasion of NMD would permit synthesis of a severely truncated PC1 protein lacking key C-terminal domains essential for ciliary trafficking, cell-cell adhesion, and mechanosensory signaling (Boletta and Caplan, 2025). Such truncated isoforms lack physiological activity and cannot compensate for the wild-type allele. Under the two-hit model, renal tubular cells carrying this germline truncating variant frequently acquire a secondary somatic loss-of-function mutation in the remaining wild-type PKD1 copy, completely ablating functional PC1 expression and triggering autonomous cyst formation.

Recent studies have provided consistent evidence regarding the influence of *PKD1* mutation type on the clinical course of ADPKD. Ali et al. (2024) recruited 22 patients with truncating and 20 with non-truncating *PKD1* mutations from Kuwait, demonstrating that renal survival was significantly shorter in individuals with truncating variants (median 51 years) than in those with non-truncating variants (median 56 years). Similarly, another recent study (Ciantar et al., 2024) analyzed 28 ADPKD patients from Malta to characterize the phenotypic effects of different *PKD1* variant classes, and yielded comparable findings: patients harboring truncating PKD1 variants developed ESRD at a younger median age (51 years) relative to those with non-truncating variants (median 58 years). Among truncating variants, the median age at ESRD onset was highest for splicing variants (63 years) and lowest for nonsense variants (49 years). Furthermore, truncating variants were associated with a higher risk of extrarenal manifestations. In our study, the identified variant was a splicing variant, which falls into the truncating category. The proband presented with multiple liver cysts, although no extra-renal manifestations were recorded in the other two affected family members. The father of the proband developed ESRD and was treated with dialysis at the age of 50 years. Thus, the clinical phenotypes observed in this family including the presence of extra-renal manifestations in the proband and the development of ESRD at 50 years in the father are consistent with the reported statistical trends for truncating *PKD1* variants. Early intervention and comprehensive care should be considered for the other affected members, which will play a significant role in enhancing outcomes and improving the quality of life.

The couples suffering from ADPKD have a 50% chance of inheriting the disease. Nowadays, the application of PGT-M enables the detection of genetic disorders in embryos and offers an alternative strategy to avoid the transmission of pathogenic mutations. During PGT-M, trophectoderm biopsy must be conducted for aneuploidy analysis and embryo haplotyping analysis. Following a successful pregnancy, prenatal genetic diagnosis is recommended—typically conducted around the 18th week of gestation—to confirm the PGT-M findings and ensure the absence of the familial variant. In this report, we described the successful application of PGT-M in an ADPKD family carrying a novel *PKD1* splicing variant (c.7489 + 5G>A). Following embryo transfer and establishment of pregnancy, prenatal diagnosis via Sanger sequencing confirmed that the fetus did not inherit the maternal *PKD1* variant. CMA performed on amniotic fluid samples revealed no pathogenic CNVs or chromosomal abnormalities. Ultimately, a healthy male infant was delivered via cesarean section at 41 weeks of gestation.

In summary, this study highlights the power of personalized reproductive medicine by translating rigorous functional genomic validation of pathogenic splicing variants into individualized reproductive care for ADPKD families. We established a complete translational pipeline spanning variant identification, *in vitro* splicing functional characterization, and clinical PGT-M implementation, forming a robust paradigm for monogenic hereditary disease prevention. By functionally validating the pathogenic *PKD1* splicing variant c.7489 + 5G>A and seamlessly integrating this molecular evidence into a successful PGT-M cycle that yielded a healthy live birth, our work underscores the critical importance of combining precise variant functional testing with advanced reproductive technology to elevate the accuracy and clinical utility of precision genetic prevention strategies for inherited renal ciliopathies.

## Data Availability

The original contributions presented in the study are included in the article/Supplementary Material, further inquiries can be directed to the corresponding author.
